# Supplementary material for: Autologous Marrow Mesenchymal Stem Cell Driving Bone Regeneration in a Rabbit Model of Femoral Head Osteonecrosis
Source: Pharmaceutics. 2022 Oct 6;14(10):2127. doi: 10.3390/pharmaceutics14102127 (PMC9610232; doi:10.3390/pharmaceutics14102127)
Supplement: Supplementary file 1 [file pharmaceutics-14-02127-s001.zip › pharmaceutics-1863150-supplementary.pdf]

pharmaceutics

Supplementary Materials

Autologous Marrow Mesenchymal Stem Cell Driving Bone Regeneration in a Rabbit Model of Femoral Head Osteonecrosis

Ilenia Mastrolia, Andrea Giorgini, Alba Murgia, Pietro Loschi, Tiziana Petrachi, Valeria Rasini, Massimo Pinelli, Valentina Pinto, Francesca Lolli, Chiara Chiavelli, Giulia Grisendi, Maria Cristina Baschieri, Giorgio De Santis, Fabio Catani, Massimo Dominici and Elena Veronesi

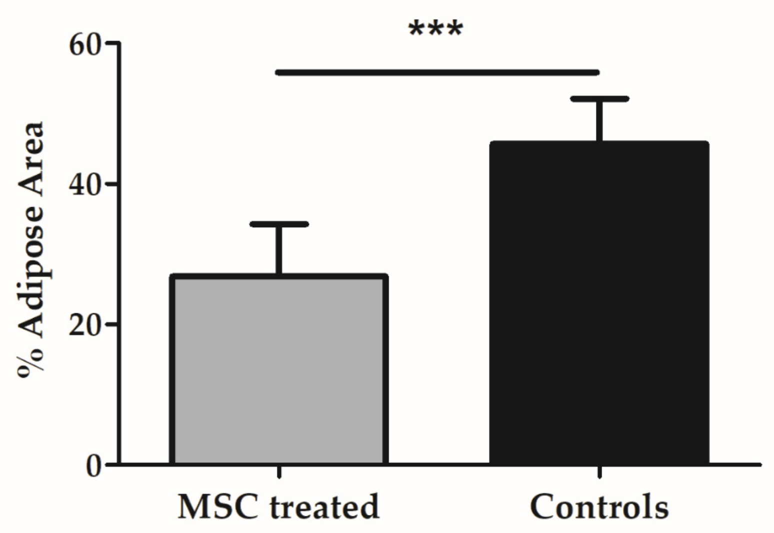

Figure S1. Quantification of adipose hypertrophy of femoral head (FH) treated with mesenchymal stromal/stem cells (MSCs) compared to the control. In the MSC-treated group, the percentage of areas of lipidic cysts is lower than control group (***$p < 0.001$).
